# 2117: Parenting, anxiety, and adaptive function in children with chromosome 22q11.2 deletion syndrome

**DOI:** 10.1017/cts.2017.244

**Published:** 2018-05-10

**Authors:** Kathleen Angkustsiri, Tony J. Simon, Paul D. Hastings

## Abstract

OBJECTIVES/SPECIFIC AIMS: Chromosome 22q11.2 deletion syndrome (22q) has a prevalence almost as common as Down syndrome. 22q is well known for medical complications, including congenital heart disease and immune dysfunction. However, children with 22q also have borderline cognitive abilities, are at high risk for ADHD and anxiety, and have poor independent living skills (adaptive function). Parenting is one modifiable factor that has been found in typically developing populations to promote independent functioning and protect against the development of anxiety disorders. This study investigates the associations between parenting, anxiety, and adaptive functioning in 22q. METHODS/STUDY POPULATION: Parent-child (ages 4–11) dyads participated in an ongoing study involving observed parenting during challenging tasks plus questionnaires of parenting, child anxiety, and child functioning. In total, 52 dyads [22q=25; typical development (TD)=27] have enrolled to date. Parents completed questionnaires, including the Parenting Styles and Dimensions Questionnaire (PSDQ), Spence Children’s Anxiety Scale, and Adaptive Behavior Assessment System for Children (ABAS-II). PSDQ dimensions of interest included Parental Psychological Control (PPC: the management of child behavior through the manipulation of emotions, expectations, and independence), Authoritative, Authoritarian, and Permissive, and the subscales of these broad dimensions. Scores were compared using *t*-tests and multiple regression models were used to investigate the relationships between 1-parenting and anxiety and 2-parenting and adaptive function. RESULTS/ANTICIPATED RESULTS: Mean age was 7.8+2.1 years. Full Scale IQ (TD: 112.3 vs. 22q: 82; *p*<0.001) and ABAS-II Global Adaptive Composite (TD: 102.7 vs. 22q: 69.2; *p*<0.001) were significantly higher in the TD group. Parents in the 22q group reported higher levels of PSDQ PPC (22q: 2.3 vs. TD: 2.1; *p*=0.06), specifically overprotection (22q: 3.7 vs. TD: 3.3; *p*=0.04), and lower Authoritative parenting (22q: 4.1 vs. TD: 4.4; *p*=0.03), across the subscales. There were no differences in Authoritarian or Permissive parenting. Children with 22q had higher Spence Total Anxiety scores (22q: 62.5 vs. TD: 47.4; *p*<0.001). Self-reported PPC and group (*R* ^2^=0.3, *F*_3,48_=8.1, *p*<0.001) predicted child anxiety with a main effect of PPC (β=16, *p*=0.02). Group tended to moderate the association between PPC and anxiety (β=−17.5, *p*<0.10), with PPC predicting anxiety for the 22q group (*r*=0.35, *p*<0.09), but not the TD group (*r*=−0.08, ns). At this time, a relationship between PPC and child ABAS-II GAC in 22q (*r*=−0.14; *p*=0.5) is not identified. DISCUSSION/SIGNIFICANCE OF IMPACT: Children with 22q are at high risk for anxiety and poor adaptive outcomes. These results suggest that parents of children with 22q use higher levels of PPC, which is correlated with increased child anxiety. These analyses also provide support for parenting interventions to improve anxiety in children with 22q and possibly mitigate the serious mental health risk in this population.

